# Curcumin potentiates antitumor activity of 5-fluorouracil in a 3D alginate tumor microenvironment of colorectal cancer

**DOI:** 10.1186/s12885-015-1291-0

**Published:** 2015-04-10

**Authors:** Mehdi Shakibaei, Patricia Kraehe, Bastian Popper, Parviz Shayan, Ajay Goel, Constanze Buhrmann

**Affiliations:** 1 Institute of Anatomy, Ludwig-Maximilian-University Munich, Pettenkoferstrasse 11, D-80336 Munich, Germany; 2 Department of Anatomy and Cell Biology, Ludwig-Maximilian-University Munich, D-80336 Munich, Germany; 3 Investigating Institute of Molecular Biological System Transfer, Tehran, 1417863171 Iran; 4 Department of Parasitology, Faculty of Veterinary Medicine, University of Tehran, Tehran, 141556453 Iran; 5 Gastrointestinal Cancer Research Laboratory, Division of Gastroenterology, Baylor Research Institute and Charles A. Sammons Cancer Center, Baylor University Medical Center, Dallas, TX USA

**Keywords:** Human colon cancer, Alginate, Metastasis, Curcumin, 5-FU, Chemosensitization

## Abstract

**Background:**

To overcome the limitations of animal-based experiments, 3D culture models mimicking the tumor microenvironment *in vivo* are gaining attention. Herein, we investigated an alginate-based 3D scaffold for screening of 5-fluorouracil (5-FU) or/and curcumin on malignancy of colorectal cancer cells (CRC).

**Methods:**

The potentiation effects of curcumin on 5-FU against proliferation and metastasis of HCT116 cell and its corresponding isogenic 5-FU-chemoresistant cells (HCT116R) were examined in a 3D-alginate tumor model.

**Results:**

CRC cells encapsulated in alginate were able to proliferate in 3D-colonospheres in a *vivo*-like phenotype and invaded from alginate. During cultivation of cells in alginate, we could isolate 3 stages of cells, (1) alginate proliferating (2) invasive and (3) adherent cells. Tumor-promoting factors (CXCR4, MMP-9, NF-κB) were significantly increased in the proliferating and invasive compared to the adherent cells, however HCT116R cells overexpressed factors in comparison to the parental HCT116, suggesting an increase in malignancy behavior. In alginate, curcumin potentiated 5-FU-induced decreased capacity for proliferation, invasion and increased more sensitivity to 5-FU of HCT116R compared to the HCT116 cells. IC_50_ for HCT116 to 5-FU was 8nM, but co-treatment with 5 μM curcumin significantly reduced 5-FU concentrations in HCT116 and HCT116R cells (0.8nM, 0.1nM, respectively) and these effects were accompanied by down-regulation of NF-κB activation and NF-κB-regulated gene products.

**Conclusions:**

Our results demonstrate that the alginate provides an excellent tumor microenvironment and indicate that curcumin potentiates and chemosensitizes HCT116R cells to 5-FU-based chemotherapy that may be useful for the treatment of CRC and to overcome drug resistance.

## Background

Conventional *in vitro* monolayer cell cultures that are frequently used for cell biology studies or for drug development are not representative of the cellular environment observed *in vivo*. In fact, the cells in monolayer cultures, by virtue of lack of tissue-specific architecture, demonstrate a dramatically reduced malignant phenotype compared to the tumor cells in *in vivo* settings [1,2]. For these reasons, the results obtained from monolayer *in vitro* cultures often cannot be translated to *in vivo* conditions. This is, in part due to the lack of an appropriate *in vitro* biocompatible microenvironment that can create and mimic a three dimensional (3D) *in vivo* metastasis situation. These limitations highlight the need for identifying and developing better *in vitro* 3D culture models of human cancer that will create a microenvironment that mimics the tumor microenvironment *in vivo* to optimize number of experiments through *in vitro* pre-testing, allowing screening of anti-metastasis drugs and mechanistic investigations under much more controllable environment [3]. Thus, the availability of adequate *in vitro* 3D culture models with better physiological relevance may have big potential as a research tool in cell biology and tumor biology.

3D alginate culture, comprising of naturally occurring non-toxic anionic polysaccharides, has been used to encapsulate a wide variety of cell types for tissue engineering and tumor research [4-6]. Indeed, several reports have suggested that cultivation of tumor cells in alginate induces cell proliferation, survival, production of extracellular matrix compounds, tumor invasion and malignancy [7-10]. Moreover, the alginate scaffolds with spheroids can be dissolved for further investigation by adding sodium citrate solution without cell damage [11]. Therefore, alginate 3D scaffolds may facilitate our understanding of tumor cell behavior, malignancy, ultimately improve the quality of *in vitro* drug screening, pre-testing clinical treatments and minimizing animal-based experiments.

The transcription factor, nuclear factor-kappaB (NF-κB), is composed of proteins with a molecular mass of 50 kDa (p50) and 65 kDa (p65) and is contained within the cytoplasm by its inhibitory subunit, IκBα. Through phosphorylation and activation, IκBα dissociates from the complex, and the NF-κB subunits freely translocate to the cell nucleus, where it regulates gene expression [12]. Several lines of evidence have shown that NF-κB plays an important role in cell survival, proliferation, invasion, angiogenesis, metastasis and chemoresistance in multiple tumor types including CRC [13,14]. Furthermore, NF-κB is constitutively activated in human CRC cells and is associated with cell progression [15,16], cell growth by inhibiting apoptosis [17], cell migration and invasion [18], cell metastasis by regulating matrix metalloproteinase-9 [19] and cell promotion by regulating cyclooxygenase-2 [20], which collectively may help mediate chemoresistance and radioresistance of tumor cells [21]. Therefore, chemopreventive agents that can suppress NF-κB activation might reduce chemoresistance and may have therapeutic potential to prevent tumor development like CRC. Curcumin (diferuloylmethane), a biologically active phytochemical component from the spice turmeric (*curcuma longa*), is one such agent. It has been demonstrated that curcumin is nontoxic in humans [22] and can block NF-κB activation and NF-κB associated gene products [23-26]. Moreover, curcumin has been shown to potentiate the cytotoxic effects of several chemotherapeutic agents such as paclitaxel, docetaxel, 5-FU and gemcitabine in malignant cells, suppressing the three major stages of carcinogenesis (*i.e*., initiation, promotion and progression) *in vitro* and *in vivo* [26-35].

5-FU is widely used as a chemotherapeutic agent for the treatment of many types of cancers and has a chemical structure similar to that of uracil and thymine [36]. 5-FU treatment blocks cancer cell proliferation and induces apoptosis by incorporation of its metabolites into DNA and RNA as a thymidylate synthase inhibitor to block dTMP synthesis [37]. High metastasis and recurrence rate of tumor cells after resection in patients is a major clinical problem, primarily due to progressive resistance of tumor cells to chemotherapeutic drugs and toxicity to surrounding healthy cells [38-40]. Indeed, it has been suggested that almost 50% of patients with CRC, may develop recurrent disease [41], indicating that no effective therapies with chemotherapeutic drugs are available to prevent metastasis and there is a great need for improved therapies and novel treatment approaches.

In the present study, we have investigated the suitability of a 3D alginate tumor model to study CRC behavior *in vitro* (the initial steps of spontaneous carcinogenesis and metastasis) and investigated in this optimized tumor microenvironment, whether the combination of curcumin and 5-FU has synergistic anti-tumor or modulatory effects on HCT116 and their 5-FU-chemoresistant counterparts.

## Methods

### Reagents and antibodies

Growth medium (Ham’s F-12/Dulbecco’s modified Eagle’s medium (50:50) containing 10% fetal bovine serum (FBS), 25 mg/ml ascorbic acid, 50 IU/ml streptomycin, 50 IU/ml penicillin, essential amino acids and L-glutamine) and Trypsin/EDTA (EC 3.4.21.4) were obtained from Biochrom (Berlin, Germany). Epon was obtained from Plano (Marburg, Germany). 5-FU and alginate were purchased from Sigma (Munich, Germany). Curcumin (BCM-95) was a generous gift from Dolcas Biotech (Landing, NJ, USA). Curcumin was prepared by dissolving it in dimethylsulfoxide (DMSO) at a stock concentration of 5000 mM and stored at −20°C. Serial dilutions were prepared in culture medium. A 100 mM stock of 5-FU was prepared in absolute DMSO and stored at −20°C. The concentration of DMSO was less than 1% of drug treatment. For treatment, 5-FU was diluted in DMEM and added to cultures to give the desired final concentration.

Polyclonal antibody against CXCR4 was purchased from Abcam PLC (Cambridge, UK). Antibodies to β-actin were from Sigma (Munich, Germany). Anti-MMP-9 was purchased from R&D Systems, Inc., (Heidelberg, Germany). Anti-phospho-specific p65 (NF-κB) was obtained from Cell Technology (Beverly, MA, USA). Alkaline phosphatase linked sheep anti-mouse and sheep anti-rabbit secondary antibodies for immunoblotting were purchased from Millipore (Schwalbach, Germany). All antibodies were used at concentrations and dilutions recommended by the manufacturer.

### Cell lines and cell culture

Human colon cancer cells (HCT116) were obtained from the European Collection of Cell Cultures (Salisbury, UK). We also generated 5-FU resistant derivatives of this cell line, referred to as HCT116R respectively, that was created by repetitive treatment of the parental cell lines to increasing concentrations of 5-FU over a 10–12 month period, as previously described [42]. Both the parental and 5-FU resistant cell lines were used to investigate the efficacy of individual and combined 5-FU and curcumin treatments. The cells were maintained in tissue culture flasks in growth medium and in a humidified incubator at 37°C in an atmosphere of 95% air and 5% CO_2_. The medium was changed every three days, and cells were passaged using Trypsin/EDTA.

### Alginate culture

A detailed description of the cell cultivation in alginate is given by Shakibaei and de Souza [4]. Briefly, the pellet of HCT116 and HCT116R cells (1 × 10^6^/ml) was resuspended in alginate (2% in 0.15 M NaCl, stirring for 1–2 h) and slowly added dropwise into a solution containing 100 mM CaCl_2_ at ambient temperature (AT). The alginate beads polymerized in the presence of CaCl_2_ after 10 min. Subsequently, the CaCl_2_ solution was removed and the alginate beads washed three times with 0.15 M NaCl solution and twice with serum-starved medium (3% FBS). Alginate beads were left untreated, treated with various concentrations of curcumin (0.1, 1, 5, 10, 20 μM), 5-FU (0.01, 0.1, 1, 10nM) or the combinational treatment of curcumin/5-FU (5 μM/0.01nM or 5 μM/0.1nM) in serum-starved medium, as previously described [26]. The medium was changed every 3 days. The cultures were grown in an incubator at 37°C with 5% CO_2_ in air.

### Phase contrast of alginate bead cultures

In order to investigate the behavior and vitality of CRC cells in alginate bead culture, whole alginate beads left untreated, treated with various concentrations of curcumin (0.1, 1, 5, 10, 20 μM), 5-FU (0.01, 0.1, 1, 10nM), or the combinational treatment of curcumin/5-FU (5 μM/0.01nM or 5 μM/0.1nM) in serum-starved medium were visualized at days 1, 3, 7, 14, 21, 28 and 35 under a light microscope (Zeiss, Germany).

### Invasion (migration) assay

HCT116 and HCT116R cell lines (1 × 10^6^/ml) were cultured in alginate beads in petri dishes for 3 weeks as described in detail above to evaluate cell invasion capacity. After an incubation time of 4–7 days, cells began to invade from alginate cultures and adhered at the bottom of the culture flask and formed colonies. During cultivation of cells in the same alginate cultures, we have isolated 3 stages of cells, (1) in alginate proliferating-, (2) active invasive- and (3) on the bottom of culture plate adhered cells, which were all taken for further investigation. Invasive cells that migrated through the alginate beads and formed adhered colonies on the bottom of the petri dish were stained with toluidine blue for 5 minutes and carefully washed two times with PBS. The number of migrated and positive stained adhered colonies were quantified and evaluated manually by counting all colonies under a light microscope (Zeiss, Germany) and visualized. This assay was repeated every 3 to 4 days until day 28 of culture. The mean number of colonies in triplicate was calculated and is reported in each bar of the graph. Each experiment was repeated at least three times.

### Western blot analysis

Whole cell extracts for western blot analysis were obtained from alginate beads, from medium (containing the emigrated, swimming spheroids) and from adhered colonies. Cells were released from alginate beads, by dissolving in 55 mM Sodium citrate solution (1,618 g Sodium citrate in 100 mL 0,15 M NaCl) for 20–30 min. Excess alginate was removed by washing twice with sterile Hanks Salt Solution and centrifugation. Medium containing emigrated spheroids was centrifuged and the supernatant discarded. Lysis buffer was added to the cell pellet obtained from alginate culture, to the cell pellet obtained from medium or directly onto adhered colonies on ice for 30 min. After homogenization and centrifugation for 30 min at 10.000 rpm, the supernatant was transferred into a new tube and stored at −80°C. Subsequently, total protein content was measured with the bicinchinonic acid system (Uptima, France) using bovine serum albumin as standard, proteins were reduced with 2-mercaptoethanol and total protein concentrations adjusted. Proteins (500 ng per lane total protein) were separated with SDS-PAGE under reducing conditions on 5-12% polyacrylamidgels. After blotting onto a nitrocellulose membrane using a trans blot apparatus (Bio-Rad, Munich), membranes were incubated with a primary antibody overnight at 4°C in blocking buffer (skimmed milk powder in phosphate buffered saline (PBS)/0.1% Tween 20), followed by incubation with the alkaline phosphatase conjugated secondary antibodies for two hours at AT. Specific antigen-antibody complexes were detected using nitroblue tetrazolium and 5-bromo-4-chloro-3-indoylphosphate (*p*-toluidine salt; Pierce, Rockford, IL) as substrates for alkaline phosphatase. Semi-quantitative evaluation was performed with densitometry (Quantity One, Bio-Rad, Munich). Specific β-actin antibody was used for the internal control to normalize the sample amounts.

### Electron microscopy

The alginate beads were fixed for 1 h in Karnovsky’s fixative followed by post-fixation in a 1% O_s_O_4_ solution in phosphate buffer. After rinsing and dehydration in ascending alcohol series, the specimens were embedded in Epon and ultrathin sections prepared with a Reichert-Jung Ultracut E (Darmstadt, Germany). Sections were contrasted with 2% uranyl acetate/lead citrate and examined under a Zeiss transmission electron microscope, Jena, Germany (TEM 10, Institute of Pharmacology, Berlin, Germany) or Jeol 1200 EXII, Akishima Tokyo, Japan (Department of Anatomy and Cell Biology, Munich, Germany).

### Quantification of apoptotic cell death

To quantify apoptosis and cells with mitochondrial changes (MC), we used the ultrathin sections of the samples and examined them with an electron microscope. The number of cells exhibiting typical morphological features of apoptotic cell death was determined by scoring 100 cells from 25 different microscopic fields per culture. The values were initially subjected to one-way ANOVA and then later compared among groups using unpaired Student’s t-test, followed by a post-hoc test to compare the parameters of each group.

### MTT assay from alginate bead culture

To evaluate cell viability of colorectal cancer cells in alginate bead culture, cells were retrieved from alginate and a MTT assay (3-(4,5-dimethylthiazol-2-yl)-2,5-diphenyltetrazolium bromide) was performed. To release the cells from the alginate, alginate beads were washed two times with sterile Hanks Salt Solution and dissolved in 55 mM sodium citrate solution. Complete dissolving of the beads was observed after 20–30 min. To remove excess alginate, cells were centrifuged, washed twice with sterile Hanks Salt Solution and resuspended in 2 ml modified cell culture medium (DMEM without phenol red, without ascorbic acid and only 3% FBS). Subsequently, 100 μl of cell suspension was distributed to a 96-well-plate, to each well were immediately added 10 μl MTT solution (5 mg/ml) and the plate was incubated for 4 h at 37°C. Finally, 100 μl of the MTT solubilisation solution (10% Triton x-100/acidic isopropanol) was added per well, and the cells incubated overnight at 37°C. Metabolically active tumor cells were evaluated through measuring the Optical Density at 550 nm (OD550) using revelation 96-well multiscanner plate ELISA reader (Bio-Rad Laboratories Inc. Munich, Germany). The values of IC_50_ (concentration which inhibited 50% of cells) was determined at each of the time intervals, by plotting data on cell viability vs silibinin concentration. The results obtained were calculated and were represented as percentage of survival relative to controls.

### Statistical analysis

Each experiment was performed three times as individual experiments with three replicates. Parameters are expressed as the mean values (+/−SD). Results were analyzed by unpaired Student’s *t-*test and by one-way ANOVA followed by a post-hoc test to compare the parameters of each group. Differences were considered to be statistically significant for *p <* 0.05.

## Results

The goal of this study was to examine whether alginate culture is suitable as a 3D tumor microenvironment to evaluate the malignant potential of CRC cells in an animal-free *in vitro* model and to investigate whether curcumin modulates and improves the effects of 5-FU on the growth of CRC cells. We evaluated the effects of curcumin on NF-κB activation, NF-κB-regulated gene products, cell growth, and invasiveness in CRC cells.

### Proliferation and invasion of CRC cells in alginate based 3D culture model mimicking the metastatic tumor microenvironment *in vivo*

Morphological investigations of encapsulated HCT116 and HCT116R cells cultured in alginate beads exhibited typical spherical shape. In all experiments, alginate beads maintained their globular morphology, did not deform and no broken beads were observed after 35 days of culture (not shown). Indeed, incubation of HCT116 and HCT116R cells either in growth medium (10% FBS) or in serum-starved medium (3% FBS) resulted in the formation of colonosphere.

**Figure 1:**
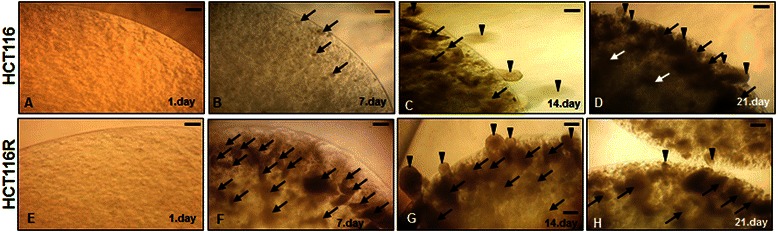
**Light microscopic demonstration of HCT116 (A-D) and HCT116R (E-H) cells (1 × 10**^**6**^**/ml) grown in alginate beads culture.** Day 1–3 of cultures **(A;E)**, encapsulated cells revealed cell aggregates, the typical spherical shape of HCT116 and HCT116R (arrows). During the following 7–10 days **(B;F)**, HCT116 and HCT116R cells formed large spheroids and were placed to the channel exterior (arrowheads) when the alginate bead surface was ruptured. With time, days 14–21 days, HCT116 **(C-D)** and HCT116R **(G-H)** cells aggregates enlarged and more and more cells migrated from the beads. x24, bar=0.2 mm in all cases.

I: Phase-contrast microscopic evaluation of HCT116 and HCT116R cells in alginate beads: HCT116 (A-D) and HCT116R (E-H) cells (1 × 10^6^/ml) were cultured in alginate beads for periods of up to 3 weeks. On day 1 of culture, the morphological appearance of the HCT116 and HCT116R cells was rounded and mainly single cells were embedded in the alginate beads (Figure 1:A, E). On day 3 of culture, HCT116 and HCT116R cells were distributed in the alginate beads and several small spheroid formations were observed (not shown). During the following days, the tumor cell aggregates grew and enlarged within the alginate beads. On day 7–10, small channels developed from tumor cell aggregates that extended into the alginate beads. The channels were filled with single or aggregates of cells. In all beads the channels developed in the same “direction”, near the surface of beads, and placed to the channel’s exterior when the alginate bead surface was ruptured, however the HCT116R cells were more proliferative and migrated earlier from alginate beads. (Figure 1:B, F). The migrated cells adopted a spheroid form at the opening of the channel to the outside of the beads. The number of detached and migrated cell aggregates of HCT116 cells was significantly increased in the periphery of the alginate beads during the following days of culture was visible by light microscopy (Figure 1:C-D, G-H).

**Figure 2:**
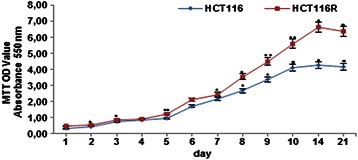
**Cell viability of HCT116 and HCT116R cells after 21 days in alginate culture.** Proliferation and viability of encapsulated HCT116 and HCT116R cells over 21 days were analyzed by MTT assay.

II: HCT116 and HCT116R cells exhibit high proliferation in alginate culture: HCT116 and HCT116R cells (1 × 10^6^/ml) were cultured in alginate beads for the indicated times, released from alginate and MTT assay was performed in a 96-well plate. Cells survived the encapsulation as spheroids and they grew fast and proliferated extensively, as demonstrated by MTT results (Figure 2). The cells proliferated rapidly, continuously and doubled their number in 3 days. However, HCT116R proliferated and grew significantly faster than HCT116 cells (Figure 2). Moreover, the proliferation of HCT116 cells reached its maximum after 10 days and HCT116R cells after 14 days in alginate beads. Taken together, these findings suggest that alginate microenvironments might be an ideal environment to study proliferation, viability and malignity of CRC cells *in vitro*.

**Figure 3:**
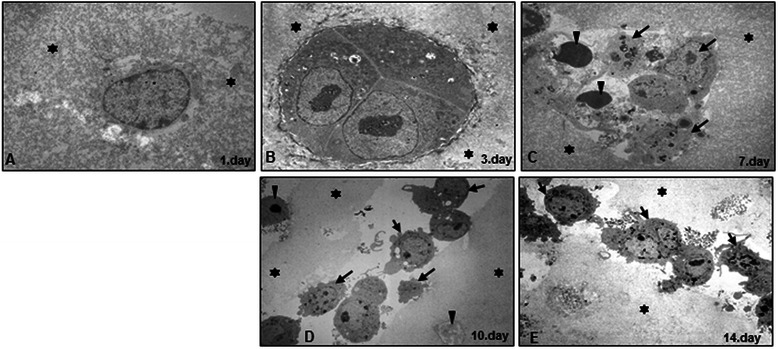
**Electron microscopic demonstration of alginate beads with HCT116 spheroids.** The HCT116 cells are embedded **(A)** and divided **(B-C)** in alginate beads (*) after 3-7days. They move apart forming more and more aggregates (arrow) and a capsule. The round to oval HCT116 cells cultured in alginate for 10-14 days **(D-E)**, showing cell aggregates within a channel (arrows), proliferating cells emigrating from the beads. Numerous cells were apoptotic and fragmented (arrowheads). x4.500.

III: Evaluation of cell viability by transmission electron microscopy: To better understand the initial steps of spontaneous metastasis behavior (proliferation, detachment, invasion) and the viability of HCT116 and HCT116R cells in alginate beads on the ultrastructure level, we performed transmission electron microscopy analysis. After one day culture period, alginate cultures of HCT116R (not shown) and HCT116 cells showed single cells and small cell aggregates embedded in alginate structure (Figure 3A). After 3–7 days (Figure 3B-C), HCT116 cells proliferated and aggregated well in alginate beads. Cells were mainly round to oval, contained a well-developed rough endoplasmic reticulum, a large golgi apparatus and other organelles or structures, such as mitochondria, small vacuoles and granules. After a culture period of 7 to 14 days (Figure 3C-E), cells started to rupture alginate structure, formed small channels with cell aggregates and migrated from the alginate (Figure 3D). The morphology of the HCT116 cells was almost unchanged in the fourth and fifth week (data not shown). During the cultivation of HCT116 cells in alginate cultures, necrosis/apoptosis occurred in a small proportion of the cells (Figure 3C-D). HCT116 and HCT116R cells revealed similar distribution profile and formation of colonospheres on the ultrastructural level.

### Malignancy and metastasis behavior of HCT116 and HCT116R in alginate cultures

**Figure 4:**
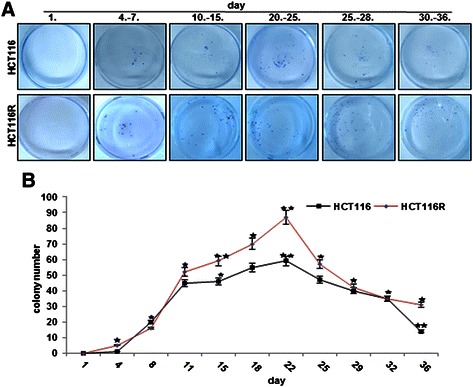
**The emigration behavior of HCT116 and HCT116R cells in alginate 3D culture.** Toluidine blue staining **(A)** and quantitative evaluation of the spheroid number **(B)** emigrated through alginate beads during the culture period from day 1–36.

Because colony formation of tumor cells is their physiologic property *in vivo*, we evaluated the long-term invasion and colony formation potential of CRC cells *in vitro*. To examine the role and effect of alginate 3D culture microenvironment on the ability of CRC cell migration and invasion, HCT116 and HCT116R cells showed fast and aggressive growth behavior regarding development of spheroids, spheroid size and spheroid distribution and grew continuously for up to 6 weeks (first stage), however the HCT116R were significantly faster (Figure 4). After an incubation time of 4–7 days, HCT116 and HCT116R cells began to invade from alginate cultures (second stage), which continued to increase in the following days and these cells adhered at the bottom of the culture flask and proliferated rapidly, formed colonies (third stage) and reached confluence 3 days later (Figure 4). As shown in Figure 4A-B, the migration and invasion capacity of HCT116R cells was more and reached a maximum after 22 days in alginate beads.

### Expression of tumor metastasis promoting factors in the 3 stages of HCT116 and HCT116R isolated cells in and from alginate cultures

**Figure 5:**
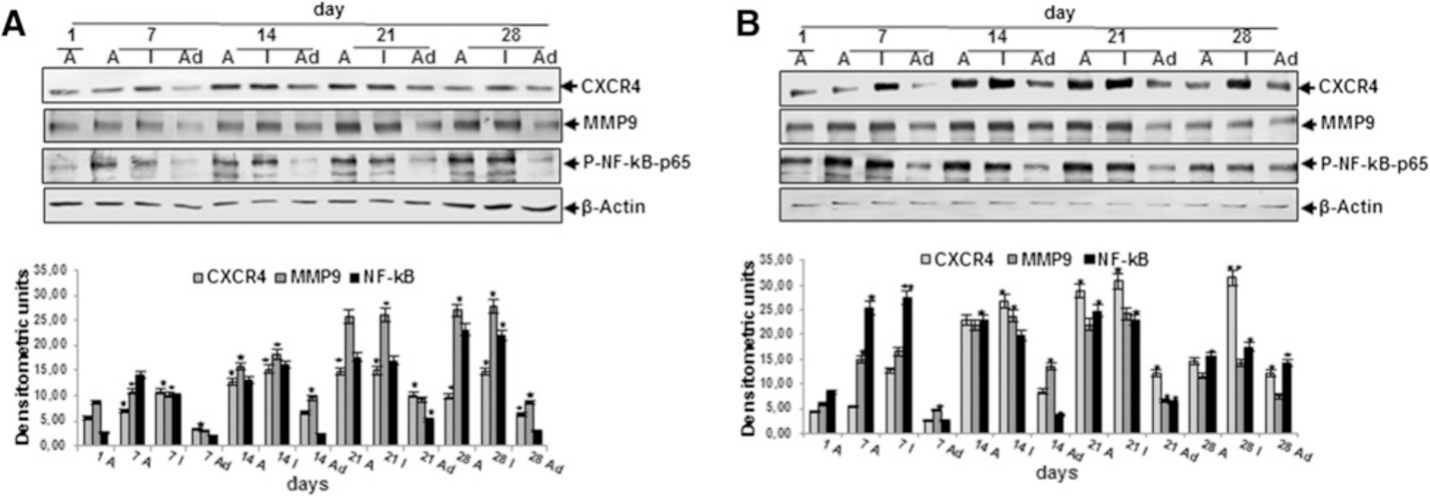
**Expression of CXCR4, MMP9 and NF-κB p65, in HCT116 (a) and HCT116R (b) cells.** Cells encapsulated in alginate beads (A) compared with migrated (invaded) (I) and adhered (Ad) cells after 1, 7, 14, 21 and 28 days of culture as shown by western blotting evaluation and was confirmed by quantitative densitometry. Western blots shown are representative of three independent experiments. The housekeeping protein β-actin served as a positive loading control in all experiments. Values were compared with the control and statistically significant values with *p <* 0.05 were designated by an asterisk (*) and *p <* 0.01 were designated by two asterisks (**).

To further characterize the malignancy and metastatic ability of HCT116 and HCT116R cells, the proliferation and metastasis-associated signaling protein expression profiles in 3D spheroids within alginate beads, in migrated (invaded) and in adherent cells was investigated. We examined tumor metastasis promoting factors (such as MMP-9, CXCR4, NF-κB) and performed western blotting analysis after 1, 7, 14, 21 and 28 days. The expression of tumor metastasis promoting factors (Figure 5) was significantly higher in HCT116 and HCT116R cells isolated from alginate beads or medium (invaded cells) compared to on the petri dishes bottom adhered cells during the whole culture period. However, it was noted that the expression of the above mentioned proteins was significantly more in 5-FU resistant cells compared with the parental HCT-116 cells (Figure 5). Densitometric evaluation of protein expression as revealed by western blot analysis was performed in triplicate.

### Curcumin potentiates the anti-tumor activity of 5-FU by apoptosis, inhibition of proliferation and colony formation of HCT116 and HCT116R cells in 3D alginate beads

**Figure 6:**
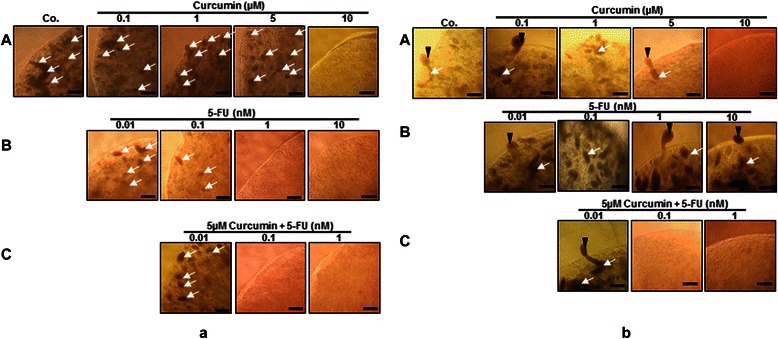
**Curcumin increases 5-FU to block the proliferation and viability of HCT116 (a) and HCT116R (b) cells (1 × 10**^**6**^**/ml) cultured in alginate beads.** Phase-contrast microscopic observations after 14 days of HCT116 cells **(a: A, B, C)** (arrows), and HCT116R cells **(b: A, B, C)** (arrows) in alginate showed the inhibition of formation of spheroids and viability of cells by curcumin, 5-FU alone and in combination of them in serum-starved medium. Samples from 3 experiments were analyzed and representative data are shown. x24, bar=0.2 mm in all cases.

**Figure 7:**
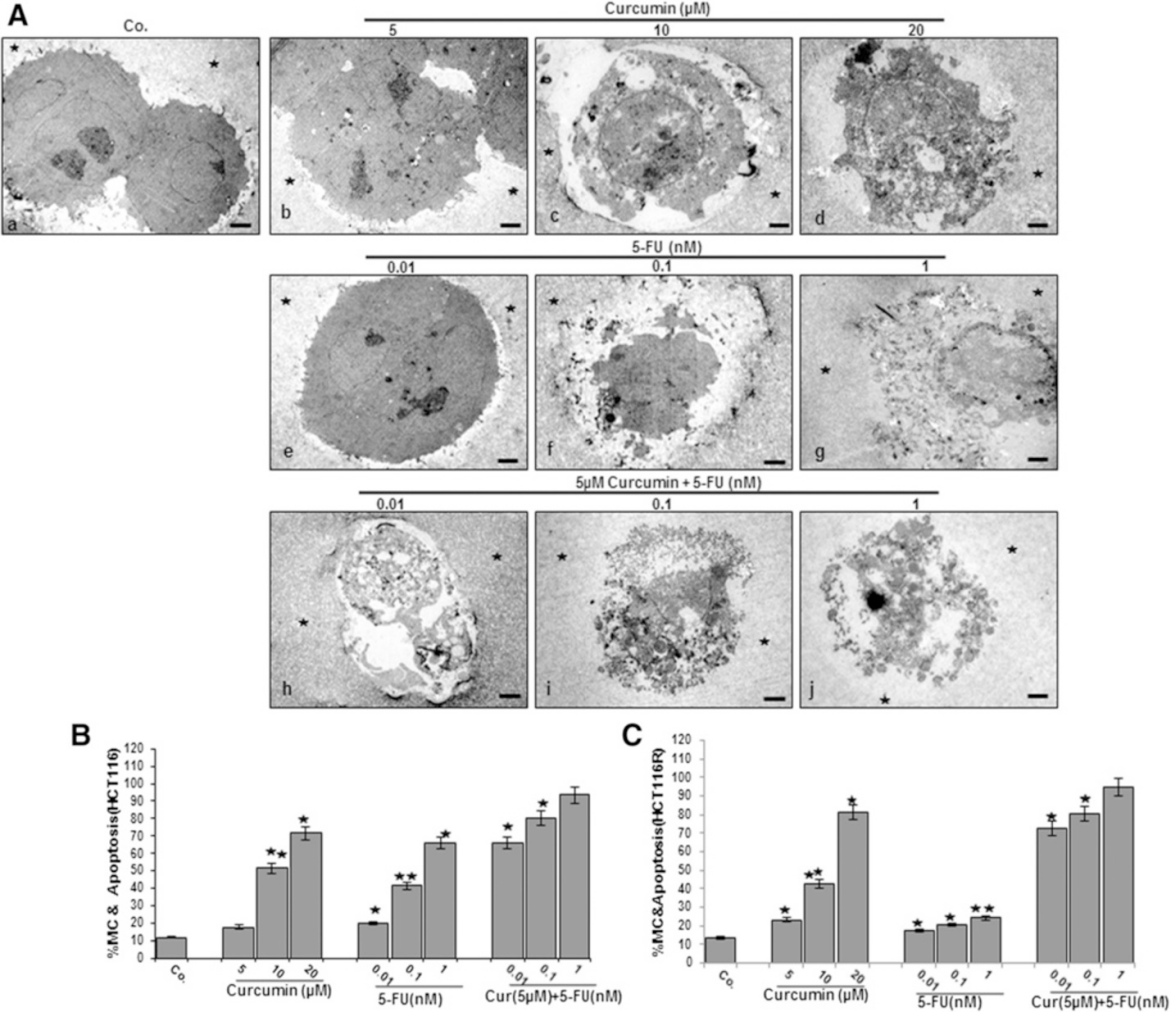
**Electron microscopic evaluation of mitochondrial and apoptotic changes after treatment with curcumin or/and 5-FU in HCT116 and HCT116R cells in alginate beads. A**: Alginate (*) cultures of HCT116 cells were either left untreated (Co.) or were treated with different concentrations of curcumin (5, 10 and 20 μM) or 5-FU (0, 0.01, 0.1 and 1nM) or a combination of curcumin (5 μM) and 5-FU (0.01, 0.1 and 1nM) in serum-starved medium for 14 days. Magnification: x5000, bar = 1 μM. **B-C**: Mitochondrial changes (MC) and apoptosis were quantified by counting 100 in HCT116 (B) and HCT116R cells (C) with morphological features of apoptotic cell death from 25 different microscopic fields and results presented are mean values with standard deviations from three independent experiments. Significant values were compared with the control and statistically significant values with *p <* 0.05 were designated by an asterisk (*) and *p <* 0.01 were designated by two asterisks (**).

To examine, whether curcumin can enhance the anti-proliferation, colony formation and invasion effects of 5-FU in 3D alginate beads, HCT116 and HCT116R cells were investigated by evaluation of spheroid formation in alginate beads after 14 days. Curcumin inhibited proliferation, viability and colony formation of HCT116 and HCT116R cells in a dose-dependent manner in alginate beads. Curcumin showed similar cytotoxic profile with a maximum effect at 10 μM in HCT116 cells (Figure 6a: A) compared with 5 μM in HCT116R cells (Figure 6b: A). It was noted that 5-FU resistant cells were more sensitive to curcumin compared to the parental HCT116 cells. 5-FU also inhibited proliferation, viability and colony formation of HCT116 cells in alginate beads and these effects were significant at a concentration of 1nM (Figure 6a:B). Interestingly, it was noted that there was little or no effect of 5-FU on HCT116R cells, even after treatment with 10nM dose (Figure 6b: B), suggesting that HCT116R cells are resistant to 5-FU, but sensitive to other chemotherapeutic agents, such as curcumin. To overcome such resistance and to increase the efficacy of 5-FU, a combined treatment was employed comprising curcumin and 5-FU. As shown in (Figure 6a: C; 6b: C) the combination dose of 5 μM curcumin and 0.1nM 5-FU had maximum effect on inhibition of proliferation and viability of HCT116 cells and HCT116R cells in alginate beads. Colony formation was completely suppressed at these combinations treatment. Interestingly, a lower concentration of 5-FU was needed in combination with curcumin to inhibit the proliferation and viability of HCT116R cells. Thus, it appeared that HCT116R cells were more susceptible than HCT116 cells to the 5-FU and curcumin combination. We next examined by transmission electron microscopy whether curcumin can potentiate the cytotoxic effects of 5-FU in HCT116 and HCT116R cells in alginate beads. Ultrastructural analysis of treated cells after 14 days showed that curcumin or 5-FU induced similar cytotoxic profile and apoptosis of HCT116 and HCT116R (not shown) cells in a dose-dependent manner. Exposure of HCT116 cells to 10 μM curcumin or 0.1nM 5-FU alone induced minimum effect on apoptosis in HCT116 cells. As shown in Figure 7A, the dose of curcumin (5 μM) or 5-FU (0.01nM) that had no effect on apoptosis alone produced synergistic apoptosis when combined significantly increased the number of apoptotic cells from 17 to 66% (5 μM/0.01nM) in HCT116 cells (Figure 7B) and from 17 to 73% in HCT116R cells (Figure 7C). Thus, it appeared that HCT116R cells were more susceptible than HCT116 cells to the 5-FU and curcumin combination.

### Cytotoxic effect of curcumin or/and 5-FU on HCT116 and HCT116R cells in 3D alginate beads

**Figure 8:**
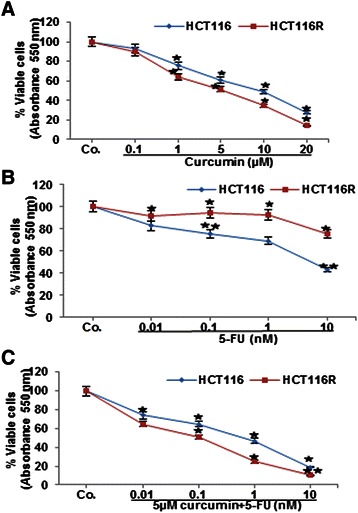
**Curcumin enhances 5-FU to inhibit cell viability of HCT116 and HCT116R cells.** HCT116 and HCT116R cells (1×10^6^/ml) were treated with different concentrations of curcumin (0, 0.1, 1, 5, 10, 20 μM) **(A)**, 5-FU (0, 0.01, 0.1, 1, 10 nM) **(B)** or HCT116 and HCT116R cells were co-treated with curcumin (5 μM) and with 5-FU in different concentrations (0, 0.01, 0.1, 1, 10 nM) **(C)** in serum-starved medium for 14 days and cell viability was measured using the MTT assay, as described under Material and Methods. Concentrations of curcumin or/and 5-FU resulting in 50% growth inhibition were indicated as individual IC_50_ values. The results are provided as mean values with standard deviations from at least three independent experiments. OD value at 100% viable cells was for HCT116 (4.4) and for HCT116R (6.7). Values were compared with the control and statistically significant values with *p <* 0.05 were designated by an asterisk (*) and *p <* 0.01 were designated by two asterisks (**).

Because colony formation of tumor cells is an important behavior to tumor cells physiology and growth *in vivo*, and to confirm the anti-proliferative effect of curcumin, we evaluated the effect of curcumin on the cytotoxic effects of 5-FU on long-term colony formation and proliferation of HCT116 and HCT116R cells. To identify the 50% cell proliferation inhibitory concentrations (IC_50_) and to understand the cytotoxic effect of curcumin or/and 5-FU on HCT116 and HCT116R cells in alginate culture, the well-established cell viability assay (MTT assay) was performed. Curcumin or 5-FU blocked the proliferation and increased cell death of HCT116 cells in a dose-dependent manner for each drug. The HCT116 cells were sensitive to curcumin or 5-FU with an IC_50_ of 9 μM or 6nM, respectively (Figure 8A, B). The HCT116R cells were sensitive to curcumin with an IC_50_ of 5 μM (Figure 8A). Moreover, to overcome 5-FU resistance and to increase the efficacy of curcumin, a combined treatment was performed. The curcumin concentrations were kept constant at 5 μM and different concentrations of 5-FU (0, 0.01, 0.1, 1 and 10nM) were used and the HCT116 and HCT116R cells were treated for 14 days. Results showed that curcumin significantly enhanced the anti-proliferative effects of 5-FU and reduced significantly IC_50_ values for 5-FU to 0.8nM in HCT116 cells and to 0.1nM in HCT116R cells, respectively (Figure 8C). These results indicate that curcumin can potentiate the anti-proliferative and colony-forming effect of 5-FU against HCT116 and HCT116R cells in 3D alginate cultures and HCT116R cells were more susceptible than HCT116 cells to the 5-FU and curcumin combination.

### Curcumin increased the 5-FU-induced inhibition of migration (invasion) in HCT116 and HCT116R cells in alginate-based 3D culture

**Figure 9:**
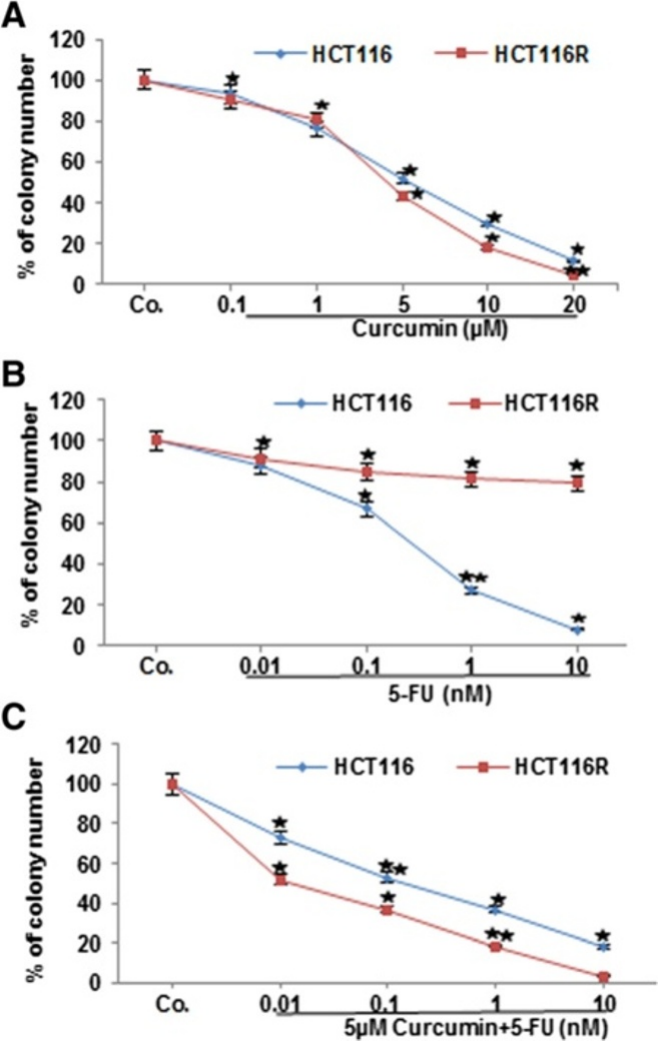
**Curcumin potentiates 5-FU to inhibit migration of HCT116 and HCT116R cells in alginate beads.** Quantification of the spheroid numbers emigrated through alginate beads after 14 days in culture. The cultures of HCT116 and HCT116R cells were treated as described above **(A-C)** and evaluated by Toluidine blue staining. Concentrations of curcumin or/and 5-FU resulting in 50% invasion inhibition were indicated as individual IC_50_ values. The results are provided as mean values with standard deviations from at least three independent experiments. Values were compared with the control and statistically significant values with *p <* 0.05 were designated by an asterisk (*) and *p <* 0.01 were designated by two asterisks (**).

Tumor cell migration *in vivo* occurs through the extracellular matrix proteins in tissues. Next, we examined whether curcumin modulates the anti-tumor effect of 5-FU against CRC migration through 3D alginate-based culture microenvironment, as an important parameter to measure cell motility for invasive and metastatic cancer cells and evaluated this by toluidine blue staining. As shown in Figure 9, treatment of the cells with curcumin alone inhibited migration of HCT116 and HCT116R cells through the alginate-based matrix in a dose-dependent manner with an IC_50_ of 6 μM and 4 μM, respectively (Figure 9A). Treatment of the cells with 5-FU alone inhibited migration of HCT116 cells through the alginate-based matrix in a dose-dependent manner with an IC_50_ of 0.4nM (p < 0.05) (Figure 9B). Interestingly, it was noted that there was little or no effect of 5-FU on HCT116R cells, even after treatment with 10nM, suggesting that HCT116R cells are resistant to 5-FU, but sensitive to other chemotherapeutic agents, such as curcumin. Moreover, to evaluate the effect of a combined treatment of curcumin and 5-FU, HCT116 and HCT-116R cells were co-treated with fixed 5 μM curcumin and with different concentrations of 5-FU (0, 0.01, 0.1 and 1nM) for 14 days. Interestingly, treatment with 5 μM curcumin significantly reduced IC_50_ values for 5-FU in HCT116 and HCT116R cells with an IC_50_ of 0.2nM or 0.01nM, respectively (p < 0.05) (Figure 9C). These results suggest that HCT116 and HCT116R cells treated with curcumin were more sensitive to 5-FU than cells treated with 5-FU alone.

### Curcumin potentiates 5-FU-induced inhibition of NF-κB (p65) activation and NF-κB-regulated gene products in HCT116 and HCT116R cells in 3D alginate beads

**Figure 10:**
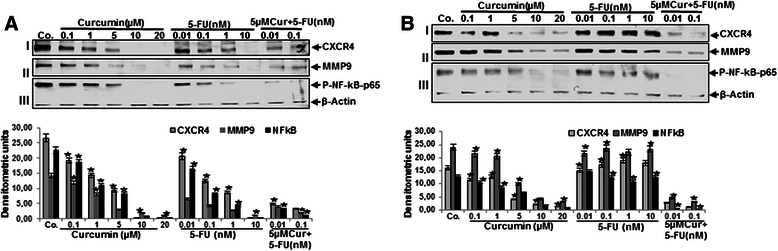
**Effects of curcumin or/and 5-FU on proliferation-, invasion-, metastatic gene products and NF-κB expression in HCT116 and HCT116R cells in alginate culture.** Curcumin increases 5-FU to inhibit the expression of NF-κB and NF-κB-regulated gene products in HCT116 **(A)** and HCT116R **(B)** cells encapsulated in alginate beads. Decreased expression of CXCR4, MMP9 and NF-κB in alginate beads after 14 days of culture was confirmed by quantitative densitometry. Western blots shown are representative of three independent experiments. The housekeeping protein β-actin served as a positive loading control in all experiments. Values were compared with the control and statistically significant values with *p <* 0.05 were designated by an asterisk (*).

To elucidate the underlying mechanism of the sensitivity HCT116R cells to the curcumin and 5-FU combination, new experiments were performed. We examined whether the effects of curcumin on CRC growth and metastasis in 3D alginate cultures was associated with the inhibition of NF-κB (p65) activation. Indeed, it has been reported that NF-κB regulates the expression of genes involved in proliferation, invasion and metastasis [13]. The alginate cultures were either left untreated or treated with curcumin (0.1, 1, 5, 10 and 20 μM), 5-FU (0.01, 0.1, 1 and 10nM) alone or were co-treated with fixed concentration of curcumin (5 μM) and with 5-FU (0.01, 0.1nM) for 14 days. As shown in Figure 10A, western blot analysis for p65 revealed that curcumin alone significantly inhibited NF-κB (p65) activation in a dose-dependent manner in HCT116 cells. The dosage of 10-20 μM curcumin almost completely suppressed the expression of NF-κB (p65) (Figure 10A,III). The combination of curcumin and 5-FU was found to be more effective than either agent alone in down-regulation of NF-κB. Therefore, we examined further the expression of the gene products which are involved in proliferation, invasion (MMP-9) and metastasis (CXCR4) (Figure 10A,I,II). Our western blot analysis results showed clearly that curcumin alone down regulated the expression of the mentioned proteins in a dose-dependent manner, but when the cells were treated with the combination of curcumin and 5-FU, the suppression effect of the mentioned proteins significantly increased (up to 80%) in HCT116 cells. Interestingly, there was little or no effect of 5-FU on HCT116R cells, even after treatment with 10nM (Figure 10B: I,II,III), suggesting that HCT116R cells are resistant to 5-FU. To overcome such resistance and to increase the efficacy of 5-FU, a combined treatment was employed comprising curcumin and 5-FU. Interestingly, co-treatment with fixed concentration of curcumin (5 μM) significantly reduced concentration of 5-FU and increased the sensitivity of 5-FU resistant cell lines. Moreover, it appeared that HCT116R resistant cells were more susceptible than HCT116 cells to the 5-FU and curcumin combination. Densitometric evaluation of protein expression as revealed by western blot analysis was performed in triplicate (Figure 10). Thus, the data suggests that the curcumin and 5-FU combination represents a potential treatment option for 5-FU resistant CRC.

## Discussion

Our results indicated that the *in vitro* alginate-based 3D culture model increased proliferation, vitality and metastatic ability of HCT116 and HCT116R cells, mimicking, at least partially, the microenvironment of CRC tumor *in vivo*. Moreover, in such a culture model, we created controllable microenvironment conditions, whereby we could understand some of the key biological behaviors of HCT116 and HCT116R cells exhibited *in vivo* in the tumor environment and investigated the anti-cancer, -metastasis activity of chemopreventive agent (curcumin) and its potentiation effect on commonly used chemotherapy agent (5-FU) against the HCT116 and HCT116R tumor cells.

The results of this study lead to the following novel findings: (1) HCT116 and HCT116R cells cultured in alginate beads were able to proliferate in colonospheres, were viable and malignant for over 21 days *in a vivo*-like phenotype. (2) During cultivation of HCT116 and HCT116R cells in the same alginate cultures, 3 stages of cells were isolated, (a) in alginate proliferating-, (b) in medium invasive- and (c) on the petri dish adhered cells. (3) The expression of tumor-promoting factors (CXCR4, MMP-9, NF-κB), which are involved with CRC proliferation, metastasis and tumor invasion were significantly increased in the alginate proliferating- and invasive cells compared to the adhered cells, however HCT116R overexpressed more proteins in comparison to the HCT116 cells. (4) Curcumin chemosensitized CRC cells and potentiated 5-FU-induced inhibition of survival, proliferation, invasion and metastatic ability of cells in a dose-dependent manner and increased more sensitivity to 5-FU of HCT116R compared to the HCT116 cells in alginate cultures. (5) The individual IC_50_ cell death of curcumin or 5-FU was approximately 9 μM or 6nM in HCT116 cells, but co-treatment with fixed concentration of curcumin (5 μM) significantly reduced concentration of 5-FU in HCT116 and HCT116R cells (0.8nM and 0.1nM, respectively) to achieve the same effect in the cells. (6) Finally, these effects were accompanied by down-regulation of NF-κB activation and NF-κB-regulated gene products.

In this study, we showed that the 3D alginate bead scaffold can serve as a matrix that realizes adequate conditions to induce cultured tumor cells towards a more malignant in *vivo-*like phenotype. Furthermore, we have developed a 5-FU resistant cell line from HCT116 cells by continuous treatment with 5-FU. These cells were resistant to 5-FU. We found that both CRC cell lines proliferated to tumor spheroids, natural behavior of many tumor cells, aggregated and migrated from the 3D-based alginate beads matrix. Moreover, the expression of proliferation- and metastasis-related proteins in HCT116 and HCT116R cells were significantly increased in the alginate proliferating- and invasive cells compared to the adhered cells on the bottom of petri dishes. However, HCT116R cells overexpressed more proteins and factors in comparison to the HCT116 cells, indicating for an increase in cancer malignancy. Thus, these results indicate the suitability of the 3D alginate bead culture as an ideal specific microenvironmental condition for CRC cells as it is significantly more representative of the tumor microenvironment than the monolayer cell culture. Furthermore, this spherical alginate-beads culture can be easily dissolved and the survived cells may be further investigated for proliferation, viability and metastatic ability of CRC cells and support the tumor microenvironment similar of that *in vivo. In vitro* cell-based experiments are expected to advance the results during early stages of the detection of remedy process by providing a cell specific response. To create such as microenvironment with stable cell differentiation in cultured cells, it is essential for obtaining and understanding the role of reliable biomedical data on cell phenotype and function [43]. Several reports have suggested that a 3D surrounding affects cell morphology, up-regulates the stem cell surface marker expression and thereby specific gene and protein expression patterns of potentially functional relevance in different tumor mammary cell lines compared with their 2D counterparts [8,44-47] and maintain their specific cell viability for a long time [48]. However, the cells in the monolayer cultures lose their differentiation and cell-specific physiological functions rapidly, seriously impairing the prognostic ability of such assays. This is to gain by culturing the cells within specific 3D scaffolds *in vitro* such as collagen types I and II, high density- or alginate cultures [4,30,43,45,49].

5-FU is widely used for the treatment of many types of cancers and is routinely employed in the management of colorectal cancer [50]. However, a serious problem with 5-FU treatment is that more than 50% of patients show resistance to 5-FU in the clinical setting, suggesting that no effective therapies with chemotherapeutic drugs are available and there is a great need for improved therapies and novel treatment approaches. Therefore, biological agents that can sensitize tumor cells to chemotherapeutic agents have great potential in treatment of cancer. Notably, several studies have shown that curcumin (diferuloylmethane), sensitizes CRC cells to 5-FU, oxaliplatin, celecoxib and to provide additional clinical benefit for patients with metastatic colorectal cancer [26,30-33,42,51-54]. We have further shown how curcumin potentiates the effects of a traditional chemotherapy agent (5-FU), by mediating its effects as anti-metastasis and -proliferative drug on HCT116 and HCT116R cell viability, proliferation and metastasis in alginate-based 3D culture model. Furthermore, curcumin alone and with 5-FU markedly reduced the capacity for colonosphere formation, migration and invasiveness of the HCT116 and HCT116R cells. We found in our *in vitro* cytotoxicity study that the individual IC_50_ of curcumin or 5-FU to HCT116 cells was 9 μM or 6nM, respectively (*p < 0.05*) and of curcumin to HCT116R cells was 5 μM. Moreover, co-treatment with fixed concentration of curcumin (5 μM) decreased significantly concentration of 5-FU in HCT116 and HCT116R cell (0.8nM and 0.1nM, respectively) to achieve the same effect in cells. Thus, the results suggest that 5-FU resistant cells are sensitive to chemotherapeutic agents, such as curcumin and the curcumin and 5-FU combination represents a potential treatment option for 5-FU resistant colon cancer.

To study the mechanism of action of the 5-FU and curcumin combination, we have first analyzed whether NF-κB transcription factor pathway was involved. Indeed, multiple pieces of evidence have shown that the NF-κB transcription factor is constitutively present and active in human colorectal tumor and that NF-κB activation was associated with a resistance to chemotherapy treatment [55-57]. Therefore, inhibition of NF-κB may be a key therapeutic target to render colorectal tumor cells susceptible to chemotherapeutic agents. Indeed, we examined the molecular mechanisms that support curcumin-mediated inhibition of CRC growth. Curcumin markedly down-regulated the activation and phosphorylation of NF-κB and NF-κB-regulated gene products that are involved in growth, proliferation, invasion (MMP-9) and metastasis (CXCR4) in CRC cells. Indeed, it has been reported that expression of CXCR4 is associated with an enhanced risk of CRC cells recurrence and survival [58]. These results are in agreement with those which showed that curcumin down-regulates NF-κB pathway through inhibition of IκBα kinase activation and IκBα phosphorylation in CRC cells [26]. Furthermore, our electron microscopy analysis demonstrated clearly that curcumin alone or/and in combination with 5-FU enhanced apoptosis in HCT116 and HCT116R tumor cells in alginate cultures. Curcumin has potentiated the anti-cancer activity of 5-FU in a synergistic inhibition of viability and proliferation against CRC cells in alginate beads *in vitro*. This is in agreement with previous investigations from our laboratory and from others which have shown that curcumin blocks the activation of NF-κB and exhibits synergistic activity with 5-FU against tumor cells [26,30,59-61]. Moreover, our results showed that curcumin potentiated the antitumor effects of 5-FU against 2 CRC cell lines *in vitro*. This finding is further in agreement with other studies showing that curcumin exhibits synergistic activity with 5-FU against HT 29 cells [60]. Du and co-workers have shown that curcumin suppresses COX-2 levels in HT 29 cells; this may account for its synergistic effects. Our results, however, indicate that curcumin blocks the expression of MMP-9 and CXCR4 expression, all of which are regulated by NF-κB and are involved in proliferation, invasion and metastasis. Thus, we suggest that this finding might have a potential implication for the prevention and treatment of colorectal tumor. Because curcumin is a component of a wide variety of fruits actively consumed by most humans and is well tolerated [22], it is preferable to examine the mechanism of action of curcumin.

It has been reported that for cancer cells in order to form metastases, the cells proliferate locally, detach from the primary environment and migrate through the tissue and survive in the circulation, adhere and proliferate at a distant organ [62]. However, it is not possible to demonstrate the important initial steps of spontaneous carcinogenesis and metastasis (local tumor growth, detachment and invasion) under *in vivo* condition. But, in our alginate model, CRC cells encapsulated in alginate beads proliferated with detachment of the cells from the alginate environment and formed CRC metastases, suggesting that this 3D model is essential and quite comfortable to recapitulate *in vitro* the sequential steps of CRC metastasis (malignancy) *in vivo*. Additionally, we have shown that curcumin increased the 5-FU-induced inhibition of HCT116R cells invasion compared with HCT116. Fifty percent of HCT116 cell invasion was blocked when 0.1nM 5-FU was added with fixed concentration of curcumin (5 μM), but a much lower concentration of 5-FU (0.01nM) was needed to achieve the same effect in 5-FU resistant cells, indicating that curcumin was more effective to sensitize the HCT116R cells than HCT-116 cells when combined with 5-FU.

## Conclusions

In conclusion, for the first time, we describe herein the monitoring of an alginate-based spherical beads culture model to provide a 3D biocompatible microenvironment for CRC cells *in vitro* for long-term cultivation. Moreover, this can improve the quality of *in vitro* drug screening, pre-testing animal-free clinical treatment, investigation of the initial steps of spontaneous carcinogenesis and metastasis. Additionally, our results suggest that curcumin sensitizes CRC cells to 5-FU, at least in part by suppressing of NF-κB signaling pathway, indicating combination of curcumin and 5-FU may be useful to overcome 5-FU resistance in CRC patients.

## Abbreviations

AT: Ambient temperature
CRC: Colorectal cancer cells
CXCR: C-X-C chemokine receptor
DMEM: Dulbecco’s modified Eagle’s medium
FBS: Fetal bovine serum
5-FU: 5-fluorouracil
MC: Mitochondrial changes
MMP: Matrix metalloproteinase
MTT: 3-(4,5-dimethylthiazol-2-yl)-2,5-diphenyltetrazolium bromide
NF-κB: Nuclear factor-κB
PBS: Phosphate buffered saline
SDS-PAGE: Sodium dodecyl sulfate-polyacrylamide gel electrophoresis
TEM: Transmission electron microscope
